# Chemotherapeutical treatment of basal cell carcinoma with bleomycin via microinfusion of the drug into the skin (MMP®)^☆^

**DOI:** 10.1016/j.abd.2022.09.009

**Published:** 2023-05-09

**Authors:** Paulo Rodrigo Pacola, Renato Roberto Liberato Rostey, Fernanda de Figueiredo Arruda Rizzo

**Affiliations:** Department of Dermatology, Hospital Universitário Júlio Muller, Cuiabá, MT, Brazil

**Keywords:** Bleomycin, Carcinoma, basal cell, Drug therapy, Skin neoplasms, Therapeutics

## Abstract

**Background:**

Bleomycin is a chemotherapeutical drug used to treat several neoplasias, including non-melanoma skin cancer; it is effective in the treatment of basal cell carcinoma (BCC) via intralesional infiltration. Transdermal drug delivery, which includes technologies such as CO_2_ Laser, Dermapen, Dermaroller and MMP®, delivers the desired medication to treat skin neoplasias and also acts in skin rejuvenation.

**Objective:**

To treat BCC lesions using bleomycin via MMP®.

**Methods:**

Ninety-eight BCC lesions in different anatomical areas were treated using MMP® technology to administer and uniformly distribute bleomycin throughout the lesion and in the established safety margin.

**Results:**

The cure rate after six months was 96.94%; and recurrences were not associated with lesion size and/or depth. Adverse effects were the expected ones.

**Study limitations:**

The follow-up time was only six months.

**Conclusion:**

This therapeutic route showed to be promising and effective.

## Introduction

Basal cell carcinoma (BCC), considered the most common skin cancer worldwide,^1^ is among the most prevalent ones in Brazil. Despite the low risk of metastases, it is a cancer with a destructive characteristic at the site where it is found. It is classified into different histopathological types, comprising low aggressive ones such as superficial and nodular, or highly aggressive ones such as sclerosing and micronodular. Different body areas classified as low-risk such as trunk and limbs, medium risk in the head and neck and high risk in the “H” zone of the face, genitals, hands and feet.^2^

The reference treatment for basal cell carcinoma is surgical excision, with the choice of conventional surgery or Mohs micrographic surgery, which will depend on the anatomical location, histopathological type, number of recurrences, immunosuppression status, comorbidities, nutritional status, and surgical size, among other factors. Among the superficial therapies, radiotherapy, imiquimod, 5-fluouracil, cryotherapy, and photodynamic therapy showed the best performance, in that order, in relation to treatment success.^3^

However, the choice of the ideal method for each patient will depend on all possible factors to be evaluated, such as age, comorbidities, anatomical area, number of lesions, access to treatment, availability of treatment in the private and public health systems, histopathological type, in addition to each physician’s practical experience. Therefore, evaluating new treatment techniques in association with established therapies, becomes interesting from the point of view of medical practice.

The application of drugs into the skin, performed through different therapeutic modalities, such as topical application, infiltration, mesotherapy, and drug-delivery (Dermapen®, Dermaroller®, CO_2_ Laser), aims at different therapeutic uses, depending on which technique and medication is being utilized. In the technique of microinfusion of a drug into the skin (MMP®), the medication is injected into the skin while it is being perforated. In this technique, the device can be adjusted, so that the needle reaches the desired level (superficial, medium, or deep dermis), and the medication is delivered at the chosen level.

Bleomycin sulfate, a medication classified as an antibiotic,^4^ antitumor or cytotoxic drug, administered by different routes (subcutaneous, intramuscular, intrapleural and intravenous), is approved by the Food and Drug Administration (FDA-USA) as a chemotherapeutic agent to treat malignancies, with squamous cell carcinoma, testicular carcinoma, lymphomas, and malignant pleural effusion as its main indications.^5^ Its use in the management of basal cell carcinoma, with intralesional administration through the skin, has also been shown to be effective in some studies.^6^

In addition to bleomycin, other more commonly used (mainly off-label) intralesional agents in the treatment of basal cell carcinoma are methotrexate (MTX), 5-fluorouracil (5-FU), and interferon, with interferon-α and 5-FU being the most widely used.^5^ There are reports of successful treatment with bleomycin in skin lesions of warts, keloids, hemangioma, keratoacanthoma, early-stage squamous cell carcinoma, and single lesions of Kaposi’s sarcoma and mycosis fungoides.^7^

Aiming to evaluate the therapeutic efficacy of this new technology employing the well-established chemotherapeutic agent bleomycin, this study proposed the treatment of basal cell carcinoma lesions.

## Materials and methods

The present study was submitted to the appreciation and approval of the Research Ethics Committee of Hospital Universitário Júlio Muller (Cep-HUJM), UFMT. This experimental clinical trial was carried out at the Dermatology Surgery outpatient clinic of the dermatology residency at HUJM. The patients were selected by convenience sample from all patients referred to the HUJM dermatology surgery outpatient clinic.

Among the patients who were examined and had the clinical-dermoscopic diagnosis of basal cell carcinoma, an evaluation was carried out of all the included patients over 18 years of age who did not meet the following exclusion criteria: lesions in high-risk areas where surgical treatment was imperative; pregnant and breastfeeding women; lesions located in areas that would compromise the application of the technique (eyelashes fringe, internal auditory canal, genital and intergluteal regions); lesions in an anatomical location where treatment would functionally impair the patient; lesions on the labial mucosa; lesions with two or more recurrences; lesions adhered to deep planes; clinical conditions that made the technique unfeasible (immunosuppression, transplanted patient); non-acceptance by the patient to be treated with this therapeutic option.

Those who met the abovementioned pre-established criteria were offered the possibility of treating the disease using this technique. Those who agreed to participate in the study signed the free and informed consent form and underwent a fusiform biopsy of the lesions to obtain diagnostic confirmation, histopathological type, biopsy thickness and tumor thickness.

**Figure 1:**
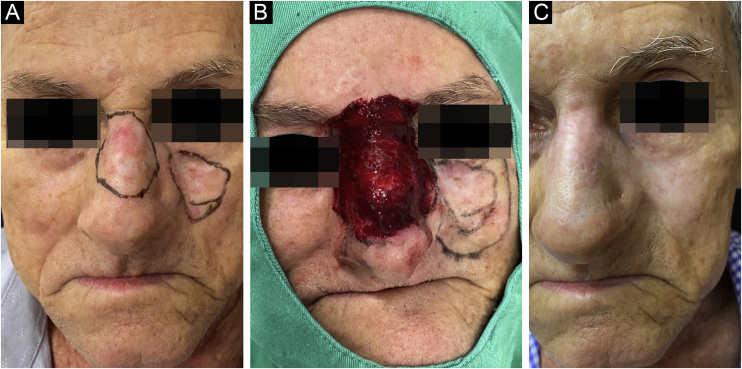
Patient with demarcation of two contiguous lesions of nodular basal cell carcinoma (A), surgical procedure, with demmarcated surgical margins, and one of the lesions in the post-treatment period with microinfusion of drug into the skin (B), and outcome after six months of treatment (C)

Once the diagnosis was confirmed, the patients followed all the precautions for conventional surgery, with lesion delimitation by dermoscopy, demarcation of the surgical margins that varied from 0.5 to 1 cm depending on the anatomical location and definition of the margins, followed by local antisepsis, placement of sterile surgical fields and local anesthesia with lidocaine and vasoconstrictor to perform the procedure without pain interference and reduction of bleeding (Fig. 1).

Lesions that were elevated above the adjacent skin were submitted to shaving so that the entire treated surface was located on the same plane and the needles reached the same depth within the lesion and the safety margin.

The intervention was performed using the Cheyenne tattoo machine and sterile disposable Cheyenne cartridges, both from MT.DERM (Berlin, Germany), and approved by ANVISA for medical use and drug delivery.^8^ In this study, Magnum Art.-Nr. E-MC17-R35LB cartridges were used, which contain 17 needles with a diameter of 0.35 mm.

**Figure 2:**
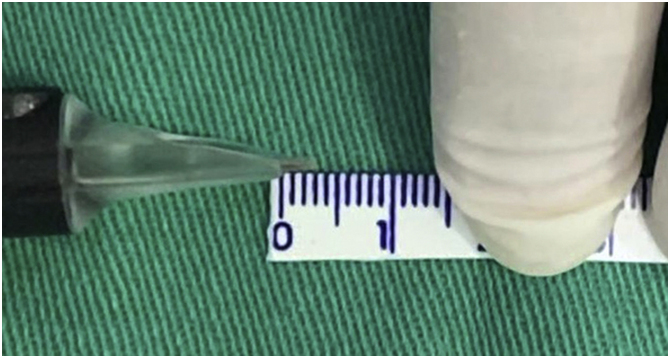
Demonstration of the needle reach when the Cheyenne device is used

Taking into account that the device has an adjustment to regulate the depth of the needles, the maximum possible depth was set as a fixed parameter to standardize the treatment and the technique, which corresponded to approximately 3 mm (Fig. 2).

The standard dilution of 15μ bleomycin sulfate was carried out in 5 mL of distilled water, resulting in 3μ/1 mL. This sterile solution was aspirated using a 30-unit B.D Ultra-fine™ sterile disposable syringe (equivalent to 0.3 mL) and transferred to the Magnum cartridge. The required amount was used according to the need for each lesion, based on the size and absorption observed during the procedure. During microneedling, the entire lesion and all predetermined safety margins were treated with at least two applications at the same point. After homogeneous microneedling, the treated area was occluded and isolated with plastic film for 12 hours. Cicaplast® or Bepantol baby® healing cream were prescribed for use until complete healing of the treated area. The follow-up appointment for the first evaluation was scheduled within a 30-day period to assess the therapeutic response and possible signs and symptoms that occurred during this period. After six months a new biopsy was performed at the site of the scar corresponding to the first biopsy.

Treated patients were followed at the outpatient clinic for a period of at least five years, aiming to assess possible late recurrences. If there was an early or late recurrence of skin cancer, it would be treated using another technique of choice, as recommended by the N.C.C.N. (*National Comprehensive Cancer Network*).

## Results

Thirty-two patients were initially included in this prospective study, from which 116 biopsies were obtained. Of these lesions, 112 had a confirmed diagnosis of basal cell carcinoma and were treated with the present technique. Two cases of actinic keratosis, one epidermal cyst, and one squamous cell carcinoma were excluded from the study.

Of the 32 treated patients, 26 returned for the biopsy six months post treatment, totaling 98 lesions that were included as the final data for statistical analysis. An active search was carried out for the six remaining patients, without success, thus 14 initially treated basal cell carcinoma lesions were excluded. All patients who completed the treatment followed the protocol of biopsies and follow-up appointments to evaluate the results.

**Figure 3:**
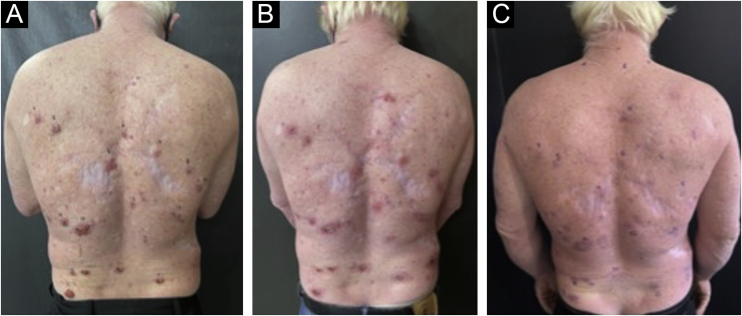
Albino patient with multiple basal cell carcinomas lesions, demonstrating pre-treatment lesions (A), lesions during the healing process after one month of treatment (B) and outcome after six months (C)

The analyzed data showed 58% female and 42% male patients. Of the study participants, three patients were albinos, with the remaining patients having phototypes I, II and III according to Fitzpatrick's classification. Of the 32 patients included in the study, two of the albino patients were the ones with the most basal cell carcinoma lesions, totaling 32 lesions. Only one patient was receiving anticoagulant treatment and had to discontinue the medication three days before the procedure. Even though this care was taken, the patient reported heavier bleeding when compared to the other patients within the first hours after the procedure (Fig. 3).

The predominance of lesions was observed in the head and neck region (48%), followed by the trunk (41%) and limbs (11%). The albino patients participating in this study showed a higher concentration of lesions in low-risk areas (trunk followed by limbs). The average size of the lesions was 3.46 cm^2^, with 0.24 cm^2^ and 33 cm^2^ respectively being the smallest and largest treated lesions (Table 1).

**Table 1:** Summary of cases, 2021

N. of basal cell carcinomas and distribution by sex	Histological type	Location
56 lesions / 12 men	Superficial 06	Cervical 03
42 lesions / 14 women	Nodular superficial 01	Face (except nasal) 24
	Nodular 80	Nasal 15
	Sclerosing nodular 05	Retroauricular 01
	Sclerosing 04	Trunk 41
	Micronodular 01	Ear 05
	Basosquamous 01	Dorsum of hands 01
		Forearms 04
		Arms 03
		Leg 01
98	98	98

Source: Chemotherapeutical treatment of basal cell carcinoma with bleomycin via microinfusion of drug into the skin (MMP®).

The 112 biopsies performed in all patients initially included in the study presented on histopathology a predominance of the nodular pattern (80.3%), followed by the mixed pattern (8%), nodular-sclerosing (7.1%), nodular-superficial (0.9%), superficial (5.4%), sclerosing (3.6%), micronodular (1.8%), and basosquamous (0.9%).

Measurements of the tumor thickness and anatomical specimen were obtained in pre-treatment biopsies. The average thickness of the specimens was 2.83 mm in the different anatomical sites, with 0.7 mm being the smallest and 6.8 mm the largest. As for the tumor lesion, the average thickness was 1.93 mm, with 0.2 mm being the smallest and 5.5 mm being the largest.

The average bleomycin dose used in the study was calculated based on the treated tumor area, and the average dose used was 2.26U (measured volume with the 30U B.D ultra-fine™ syringe) equivalent to 0.0266 mL/cm^2^ of the diluted bleomycin solution. The mean volume per patient was 7.8μ, or 0.078 mL of solution, ranging from 3μ to 30μ of solution.

**Figure 4:**
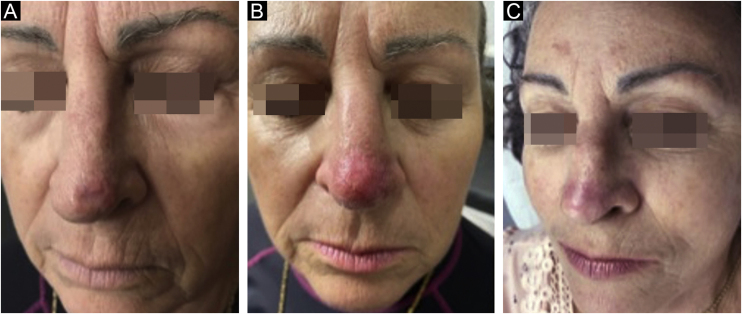
Sclerosing nodular basal cell carcinoma lesion on the nasal tip pre-treatment (A); outcome after one month (B) and after six months of treatment (C)

Control biopsies performed after six months of treatment showed scarring with no neoplasm in 96.9%, and only 3.1% had residual disease, two nodular and one nodular-sclerosing basal cell carcinoma. When analyzing the persistent lesions, it was observed that the basal cell carcinoma with the largest area (8.4 cm^2^) was treated with an average dosage of 1.67μ of bleomycin, lower than the average of the study (2.26μ). The carcinoma with a mixed histopathological pattern showed tumor thickness close to that reached by the needle (3 mm), which could be the reason for persistence (Fig. 4).

At the first follow-up appointment after treatment, the patients were asked about signs and symptoms that could have occurred as a result of the procedure. Twenty (62.5%) patients complained of pain in the first hours after the procedure, 18 (56.3%) patients complained of pruritus during the healing process, 20 (62.5%) patients referred bleeding in the first 12 hours, 2 (6.3%) patients complained of residual hyperchromia, 6 (18.8%) patients of thickened scars and 9 (28.1%) patients of atrophic scars. Overall, the side effects were well tolerated, and no patient was dissatisfied with the obtained result, as it was a skin cancer treatment.

## Discussion

Bleomycin, originally isolated from the fungus *Streptomyces verticillus*,^9^ is often used as an antitumor agent. The presence of bleomycin hydrolase in tissues inactivates bleomycin by hydrolyzing the amide bond in the beta-aminoalanine-amide portion. The enzyme deficiency in the lungs and skin^10^, ^11^ leads to a higher concentration of the drug in the skin,^12^ and bleomycin-induced toxicity predominantly occurs in these organs. In systemic treatments with bleomycin, the medication may induce scleroderma-like fibrotic lesions in the skin when used in high doses.^13^

In dermatology, bleomycin is primarily used as an intralesional treatment, and the dose usually does not exceed 2U to 6U per session^4^, ^14^ (OBS: The bleomycin dose was converted into US Pharmacopeia Units [U]: 1U represents 1 mg [per potency] or 1000 International Units [IU]).^15^ Doses as low as 0.01U/mL induced a measurable clinical inflammatory response, and maximum induration and inflammation were reached with a dose of 0.1 U/mL.^16^ In the present study, using the technique of microinfusion of medication into the skin, 3U to 30U were used, that is, 0.03 mL to 0.3 mL.

Intralesional bleomycin injection leads to direct and indirect cytotoxicity of keratinocytes and eccrine epithelium.^17^ Immunocytochemistry demonstrates the fixation of bleomycin in the tissue at all levels of the epidermis except the basal layer and most prominently in the stratum granulosum (granular layer).^18^ The reactions observed after intralesional administration include gangrene, onychodystrophy, Raynaud’s phenomenon, scleroderma, and flagellate erythema.^19^, ^20^, ^21^, ^22^ After injections of bleomycin into the skin, local reactions and transient symptoms may occur, such as erythema, edema, darkening of the skin, eschar formation, pain, and pigmentary changes.^23^, ^24^

Different from the intralesional infiltration that deposits the medication “in bolus”, the uniform distribution and on the same plane achieved by microinfusion, showed initial reactions of bleeding in the first hours after the procedure and mild pain. Subsequently, crust formation occurred, followed by atrophic scar formation. After one month, some phototype III patients showed mild hyperpigmentation. Although the patients showed an atrophic scar in the first month after the procedure, which was the predominant cicatricial result, hypertrophy appeared later in some patients in the treated lesions located in the central region of the face. This reaction was possibly due to the fact that this study was carried out during a period when the use of a face mask was required to prevent COVID-19 infection.

Pharmacologically, bleomycin has been shown to block the cell cycle in G2,^25^ to cleave single- and double-stranded DNA,^26^ and to degrade cell RNA.^27^ This is due to binding to DNA by electrostatic attraction.^28^ Bleomycin forms a complex with metal ions such as Fe (II), which is subsequently oxidized to Fe (III), resulting in the reduction of oxygen to free radicals. These free radicals cause DNA breakage, ultimately leading to cell death.^29^, ^30^ The antitumor activity of bleomycin is due primarily to its capacity to cleave DNA. The positively charged amino terminal group of bleomycin is electrostatically attracted to the negatively charged phosphate group on the DNA, and the planar bithiazole group is intercalated in the double-stranded DNA. The DNA strand is cleaved by the activated oxygen molecule in the bleomycin ferrous complex.^31^

Moreover, bleomycin is known to induce apoptosis *in vitro*.^32^ This pro-apoptotic effect of bleomycin is mentioned as another of its antitumor properties. By affecting protein synthesis, bleomycin causes a series of biochemical changes that lead to apoptosis and total epidermal necrosis. These effects are dose-dependent, and frequent injections of low doses of bleomycin are reported to be the most effective.^33^

Another possible mechanism underlying the efficacy of bleomycin is the induction of tumor necrosis factor (TNF).^16^ Tumor necrosis factor is also known to upregulate the expression of intercellular adhesion molecule-1 (ICAM-1), endothelial leukocyte adhesion molecule-1 (ELAM-1), and vascular cell adhesion molecule-1 (VCAM-1), to induce tissue factor-like procoagulant activity in human endothelial cells, and to cause hemorrhagic tumor necrosis.^34^

Intralesional infiltration of bleomycin supplemented with lidocaine, in addition to the anesthetic effect, increases the intracellular uptake of this chemotherapeutic antibiotic. Lidocaine acts as a local anesthetic and increases the uptake of hydrophilic chemotherapeutic agents, such as bleomycin, through a membrane-stabilizing effect.^6^

Templeton et al.^16^ evaluated the response elicited by bleomycin when applied intradermally to normal human skin and observed that the inflammatory reaction started ten to 12 hours after the injections, peaked at 24 to 48 hours, and often lasted more than eight to ten days. What histopathological changes were observed superficially and deeply in the skin? Perineural inflammation of varying density was present in four of the five initial biopsy specimens. Immunofluorescence microscopy demonstrated that bleomycin-treated skin showed a marked increase in HLA-DR in epidermal keratinocytes, focal induction of ICAM-1 in epidermal keratinocytes, and upregulation of ICAM-1 expression in superficial dermal blood vessels. The time course of the inflammatory response was similar at injection sites of all bleomycin concentrations.^16^ In the present study, it was clinically observed that the treated lesions showed an inflammatory process accompanied by crust formation within 48 hours, and this inflammatory process remained in a regressive manner for the first 30 days, at which time a scar, pinkish erythema and residual desquamation were observed.

The literature review showed higher rates of cure of basal cell carcinoma with intralesional infiltration of bleomycin when compared to other agents such as MTX, 5-FU, and interferon; however, the sample size was smaller for the bleomycin group in comparison with the other groups.^35^

**Figure 5:**
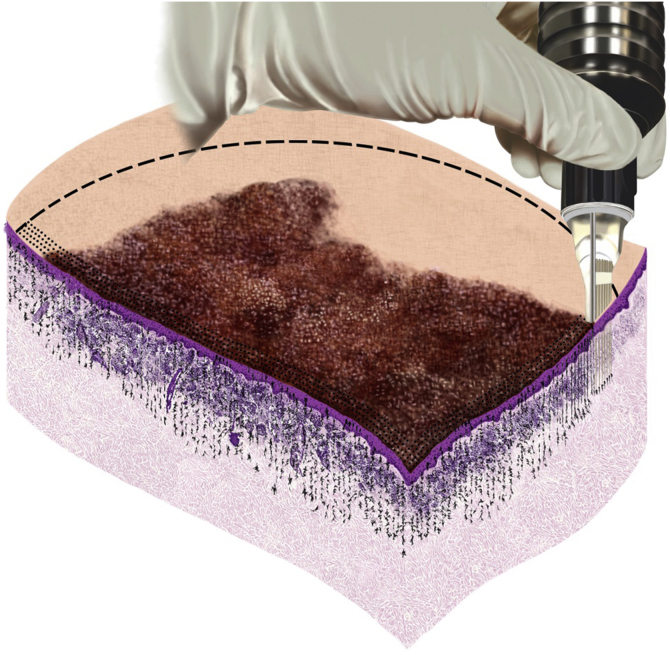
Lesion with demarcated surgical margin, cartridge needles adjusted to maximum depth, and bleomycin evenly distributed throughout the lesion and safety margin

Among drug-delivery techniques used in the treatment of skin neoplasias, MMP® has been shown to be effective. Its main characteristic is the uniform injection and distribution of the medication that occurs simultaneously with the perforation, which can have its depth regulated according to the neoplasia to be treated, the anatomical area where the lesion is located, and the medication chosen for each situation. In the present study, which assessed basal cell carcinoma lesions, the treatment was performed according to the surgical protocol for the delimitation of the lesion and surgical margins, homogeneous application, and distribution of the medication, as shown in the illustrative model (Fig. 5).

The tattooing device used in MMP® uses solid needles and the drug surrounds the outer surface of the microneedles. The friction generated by the rapid penetration of these needles into the dermis creates shear stress and a turbulent swirl of the drug, which increases its dermal diffusion.^36^ Therefore, when choosing to use a hand-held hollow hypodermic needle or a tattoo machine, physicians should be aware that these interventions produce different patterns of drug diffusion into the dermis. Among the different needle options available, the texturized (Tx) tattoo needle was selected in the present study, a characteristic that damages the skin a little more.

The use of the microneedling technique with a tattooing machine under aseptic surgical conditions may also minimize reported adverse effects reported in other tattooing procedures. It must be considered that bleomycin also has an antibiotic action, which makes the procedure even safer in terms of infection risk.

When analyzing the pharmacokinetics of drugs injected into the skin, regardless of the use of syringes, tattooing devices, or CO_2_ fractional lasers, the authors believe that the systemic absorption of the drug upon delivery is undeniable, although there may be route differences in the systemic absorption (bloodstream or lymphatic), in addition to the chemical nature, the molecular weight of the drug^37^ and the volume used. It has been even shown that tattooing pigments can migrate to regional lymph nodes because ink particles are able to move through lymphatic vessels.^38^ In the present study, although no dose or investigation was carried out regarding the absorption of bleomycin, there were no complaints of systemic symptoms or lymph node enlargement by the patients, possibly due to the low doses used.

As for the histopathological profile of tumors in the present study, it is consistent with data found in the literature, where the predominant histopathological type is low-risk basal cell carcinoma (nodular and superficial). Although in this sample the proportion of affected women was higher (58%) than men, men individually showed a greater number of lesions than women. In the particular context of this study, albino patients were the most affected.

Consistent with the literature is the presence of a mixed sclerosing pattern as the most frequent in women, located in the head and neck region.^39^ The mean tumor thickness found in the treated lesions was 1.93 mm, compatible with the maximum mean thickness of 1.76 ± 1.03 mm (range 1–9.5) demonstrated by Amici et al. ^40^

The use of bleomycin via microinfusion of the drug into the skin showed to be effective in the biopsy results obtained after six months of treatment. It is known that the ideal time for follow-up is at least five years and, during this interval, the statistical data may suffer some alterations. For this reason, the patients will remain on outpatient follow-up.

Although advanced cases were excluded and those that were known to require a surgical approach, either due to the extent of the disease or the compromised structures or the infiltrative aspect of the lesion, the present study included and treated lesions with a high-risk histopathological pattern according to the N.C.C.N and also located in high-risk areas of the face so that the therapeutic efficacy of the technique could be evaluated. It was also demonstrated that the medication linked to the technique showed to be complementary and safe to be used on the face.

In addition to its efficacy, the technique showed to be appropriate and sometimes advantageous in the simultaneous treatment of multiple lesions in the same patient, a situation that is unfavorable for the conventional surgical procedure. In clinical practice, the correct indication for this technique should be based on the N.C.C.N, on tumor thickness, which can be evaluated in the pre-procedure biopsy, and the experience of each physician, taking into account each patient and their peculiarities.

## Conclusion

Treatment with bleomycin via microinfusion of the drug into the skin showed to be effective for the treatment of basal cell carcinomas over a period of six months. It is a technique of medium complexity and easy reproducibility, and very advantageous for the treatment of multiple lesions. Longer follow-up will be necessary to consolidate the long-term results.

## Financial support

None declared.

## Authors’ contributions

Paulo Rodrigo Pacola: Approval of the final version of the manuscript; design and planning of the study; drafting and editing of the manuscript; collection, analysis and interpretation of data; effective participation in research orientation; Intellectual participation in the propaedeutic and/or therapeutic conduct of the studied cases; critical review of the literature; critical review of the manuscript.

Renato Roberto Liberato Rostey: Statistical analysis; approval of the final version of the manuscript; effective participation in research orientation; critical review of the manuscript.

Fernanda de Figueiredo Arruda Rizzo: Collection, analysis and interpretation of data.

## Conflicts of interest

None declared.
